# Cryopreservation Does Not Affect the Clinical Pregnancy Rate of Blastocysts Derived from Vitrified Oocytes

**DOI:** 10.1038/s41598-022-12992-x

**Published:** 2022-05-27

**Authors:** Hadi Ramadan, Tarita Pakrashi, Andrea R. Thurman, Kimball O. Pomeroy, Gerard Celia

**Affiliations:** 1 Eastern Virginia Medical School, 825 Fairfax Ave, Norfolk, VA 23507 USA; 2 Shady Grove Fertility Jones Institute, 601 Colley Ave, Norfolk, VA 23507 USA; 3 The World Egg and Sperm Bank US, 7826 East Evans Road, Scottsdale, AZ 85260 USA; 4 Virginia Commonwealth University, 1101 East Marshall Street, Richmond, VA 23298 USA

**Keywords:** Reproductive disorders, Infertility

## Abstract

Vitrified, or “frozen”, donor eggs can either be fertilized and cultured for fresh transfer (group 1), or fertilized and cryopreserved for transfer in a “frozen embryo transfer” cycle (group 2). This study compared the pregnancy rates between the two groups. Frozen donor egg cycles (N = 1213) were analyzed at the World Egg Bank. The outcome studied was clinical pregnancy rate. Cycles included only single embryo transfers (ET) without preimplantation genetic testing (PGT). A total of 600 cycles met the inclusion criteria. Group 1 included 409 cycles and group 2 had 191 cycles. There was no statistical significance in clinical pregnancy rate between the two groups (38.63% vs 32.46%, p = 0.14). Mean embryo age was higher in group 2 (5.1 vs. 5.4 days, p < 0.01). The compounding effect of vitrification when applied to two distinct stages (oocyte and embryo), has not been studied. When comparing the two groups, we found no difference in pregnancy rate. However, there was a trend towards fewer pregnancies in group 2. A larger study should be done to determine the validity of this result (Ramadan et al. in Fertil Steril, 2020).

## Introduction

Egg freezing has become increasingly successful since its beginning in 1983^1^. It has offered an avenue to those who are unable to conceive with their own eggs to be able to do so using donor eggs. The process involves thawing the donor egg, fertilizing it, then either transferring the resulting embryo or freezing the blastocyst and transferring it in a future cycle. There are multiple reasons why the embryo would be frozen to be transferred later and not immediately transferred fresh. One reason is that eggs are thawed and fertilized in batches, which means that some embryos get transferred and some are vitrified and preserved. If there are supernumerary embryos available for cryopreservation, they may be used for a future pregnancy. Another reason is asynchrony. Despite progress, pregnancies have been hindered by the problem of asynchrony^2^. The dilemma of asynchrony is often faced by fertility specialists. In short, on the day of transfer, if the blastocyst is not mature, transfer is postponed one day, but this comes at a price of having a less receptive endometrium^2^. This results in fewer live births as described by Shapiro et al.^2^. Some physicians attempt to freeze the mature blastocyst and transfer it in the next cycle to a receptive endometrium. There is concern that a second freeze might be detrimental to the embryo.

In 2015, Wang et al.^3^ found that freezing yields a higher pregnancy rate than fresh transfers. Taylor et al.^4^ found no differences in pregnancy rate when embryos are vitrified and warmed twice, however some studies^5–7^ have found negative outcomes (lower clinical pregnancy and live birth rates) after repeat vitrification/warming of blastocysts i.e. a second vitrification in the protocol before transfer.

Our study, however, is different. Our sample is frozen donor eggs which undergo thawing, fertilization, and then the embryo itself is frozen. There is no data whether this second freeze has comparable pregnancy rates to transferring a “fresh” blastocyst in the first cycle. Our objective is to compare the two methods’ pregnancy rates. The results from this study can have an impact on transfer protocols and guidelines when it comes to frozen donor eggs.

## Methods

This was a retrospective chart review at the World Egg Bank in Arizona. Eastern Virginia Medical School Institutional IRB approval was requested (IRB # 20-01-NH-0013). The letter issued stated that the Human Subjects' Protection Program (HSPP) has reviewed the request for our project and has determined that this project does not involve human subjects and therefore does not meet the definition of Human Subjects Research. As such it was not subject to IRB review. All data was de-identified and all HIPPA identifiers were removed before the data was retrieved and analyzed. All methods were performed in accordance with the guidelines and regulations of the IRB and the nature of the study. Data collected was restricted to frozen donor eggs. In this bank, oocytes were vitrified according to the protocol provided by Cryotec (Reprolife). Briefly, ova were exposed to Equilibration Solution for about 15 min followed by exactly 60 s in Vitrification Solution and then placed on Cryolock (Biotech, Inc) carriers and immersed in liquid nitrogen. Cycles analyzed were between December 2013 and June 2019. Data points collected included embryo age, number of embryos transferred, number of embryos vitrified, preimplantation genetic testing, pregnancy outcome, and whether the embryo was transferred after fertilization or frozen and then transferred later. The primary outcome studied was Clinical Pregnancy Rate (CPR), defined as having gestational sac(s) within the uterine cavity when examined by ultrasound^8^. Data on 1213 cycles was collected. Data was analyzed and 600 cycles remained after excluding cycles which had preimplantation genetic testing (PGT) (N = 8), multiple embryo transfers (N = 139), and any outliers or contradictory numbers or missing information (N = 466). Cycles undergoing PGT and multiple embryo transfers were excluded due to the rarity of both when it comes to the use of frozen donor eggs.

The data was analyzed using the Chi-squared test for categorical variables and the Wilcoxon-Mann–Whitney test for non-parametric continuous variables. P values < 0.05 were regarded as statistically significant. SAS version 9.4 was used for the analysis.

## Results

600 cycles met the inclusion criteria (Single embryo transfer, no PGT). Of those 600, 409 cycles (68%) belonged to the group that started with a frozen donor egg, thawed, fertilized, and transferred. This group was labeled as group 1. The rest, 191 cycles (32%), belonged to the group that went through the same process as group 1, however was vitrified after fertilization, then transferred later. This group was labeled group 2 and will be referred to as such throughout this paper (Table 1).

**Table 1:** Warming type, age, and pregnancy distribution.

**Warming type**	
Egg warming i.e. fresh (Group 1)	409 cycles (68%)
Embryo warming i.e. refrozen (Group 2)	191 cycles (32%)
**Embryo age**	
5 days	515 (86%)
6 days	77 (13%)
7 days	8 (1%)
Mean age	5.1 ± 0.4 days
**Pregnancy**	
Yes	220 (37%)
No	380 (63%)

As for embryo age, 515 (86%) were transferred on day 5, 77 (13%) on day 6, 8 (1%) on day 7. The mean age of the embryos was 5.1 ± 0.4 days (Table 1).

Of the 600 cycles, 220 (37%) achieved clinical pregnancy, and 380 (63%) did not (Table 1). The mean embryo age in the pregnant group was 5.1 ± 0.4 days versus 5.2 ± 0.4 days in the other group (p = 0.58).

When comparing the CPR between group 1 and group 2, it was found that it was 38.63% in group 1 versus 32.46% in group 2. Although group 1 had a higher CPR, this result was not statistically significant with p = 0.14 (Table 2).

**Table 2:** Clinical Pregnancy rate in groups 1 and 2.

	Positive clinical pregnancy	P value
Egg warming i.e. fresh (Group 1)	158 (38.63%)	p = 0.14
Embryo warming i.e. refrozen (Group 2)	62 (32.46%)	

When comparing embryo age, which is the mean age of all embryos in each group, group 1 had a lower embryo age at 5.1 days versus group 2 at 5.4 days. This difference was statistically significant with p < 0.0.1 (Table 3).

**Table 3:** Embryo age in groups 1 and 2.

	Embryo age (days)	P value
Egg warming i.e. fresh (Group 1)	5.1	p < 0.01
Embryo warming i.e. refrozen (Group 2)	5.4	

## Discussion

These results have an impact on several levels. When comparing the CPR, statistically, there was no difference between group 1 and 2. This indicated that the second vitrification in the process had no effect on pregnancy potential. Applied to the clinical setting, the physician would not be bound by the synchrony or delay of blastulation, making it an easier decision to freeze the embryo and transfer later. Although this type of study has not been undertaken before, the results are in conjunction with Taylor et al.^4^ being that repeat vitrification did not affect pregnancy rate. The difference is that in our study, the egg undergoes vitrification, then the embryo. In Taylor et al.’s study, it is the embryo that is vitrified in both steps. Other studies^5–7^ have shown negative outcomes with repeat vitrifications of embryos. Zheng et al.^6^ showed similar pregnancy rates with lower live birth rates in the twice vitrified group. A case could be made, though, that in our study, there was a downward trend of the clinical pregnancy rate (38.63% 32.46%). This trend might be significant with a larger sample size which would change the clinical decision making.

As for the embryo age, group 2 had a significantly older embryo sample than group 1. This is most likely because when choosing which embryo to transfer, clinics tend to transfer the matured blastocysts on day 5 after several days of uterine preparation with progesterone. As discussed earlier, if the transfer happens later than intended, this will cause asynchrony and lower pregnancy rates^2^. Clinicians would then avoid transferring any embryos that have not blastulated by day 5, wait until day 6 and 7, then freeze them when they mature for later cycles. This would explain why group 2 has a significantly a higher mean age than group 1.

Our study is limited by its retrospective nature. In addition, several covariates were not available to add to our analysis which limits the applicability of our results. The strength of this study lies in the fact that it has never been done before and this result is novel in the clinical setting. We had a large sample size (N = 600) which gave power to the results discussed. The data was analyzed in a separate institution than the egg bank itself, eliminating any bias.

In conclusion, our study shows that frozen donor eggs undergoing a repeat vitrification process has no effect on the clinical pregnancy rate compared to “fresh” transfers. However, a prospective study, with collection of more covariates, must be done to prove these results to apply them to the clinical setting.

## Supplementary Information

Supplementary Information.

## Data Availability

The datasets generated during and/or analysed during the current study are available from the corresponding author on reasonable request.
